# Editorial: Alcohol-associated liver disease—From pathogenesis to treatment

**DOI:** 10.3389/fphys.2022.1060812

**Published:** 2022-10-20

**Authors:** Kusum K. Kharbanda, Ashwani K. Singal, Sebastian Mueller, Natalia A. Osna

**Affiliations:** ^1^ Research Service, Veterans Affairs Nebraska-Western Iowa Health Care System, Omaha, NE, United States; ^2^ Department of Internal Medicine, University of Nebraska Medical Center, Omaha, NE, United States; ^3^ Department of Biochemistry and Molecular Biology, University of Nebraska Medical Center, Omaha, NE, United States; ^4^ Sanford School of Medicine, University of South Dakota, Vermillion, SD, United States; ^5^ Center for Alcohol Research, University of Heidelberg, Heidelberg, Germany

**Keywords:** alcohol, liver disease, pathogenesis, therapeutic targets, biomarkers

The Topic “*Alcohol-Associated Liver Disease–From Pathogenesis to Treatment*” proposed by the Frontiers in Physiology has attracted a total of 20 articles on preclinical and clinical studies that were covered in 16 original research manuscripts, 2 brief research reports, 1 review and 1 systematic review. Overall, these articles provide a better understanding of the molecular mechanism(s) that mediate the development and progression of alcohol-induced liver pathology, as well as identifying new biomarkers, therapeutic targets and promising novel therapeutic agents to prevent, manage, or reverse the disease progression. In this editorial, we as guest editors provide a summary of all the articles published in this Research Topic.

There were four original papers that described pathogenic mechanism of disease progression. An article by Valchin et al. deliberated on the role of the molecular circadian clock disruption in liver pathology. They showed that alcohol consumption disrupts 24 h rhythms in mRNA levels of multiple clock and lipid metabolism genes in the liver causing small droplet macrosteatosis. Using liver specific knockouts, these authors concluded that the liver clock is important for maintaining temporal control of hepatic lipid metabolism and suggested that circadian clock disruption may be an important risk factor in the pathogenesis of ALD. Wang et al. using a rat model demonstrated that sustained sleep deprivation caused an elevation of liver enzymes and plasma lipids levels which was associated with hyper activation of the sympathetic nervous system. The authors further showed that insomnia-induced hepatic steatosis could be abrogated with pharmacological ablation of the hepatic sympathetic nerves. Furthermore, the treatment of insomnia with estazolam inhibited sympathetic activation and reduced hepatic steatosis. The article by Dou et al. highlighted the key role of long non-coding RNAs (lncRNAs) in the development of ALD pathogenesis. The authors revealed that the five lncRNAs downregulated in a mouse model could serve as novel biomarkers of ALD. The authors further demonstrated that the most downregulated lncRNA, lnc_1700023H06Rik, played a pivotal role in lipid deposition. A manuscript by Li et al. showed that excessive drinking can induce miR-378b, which disrupts insulin signaling by targeting insulin receptor and p110α genes to downregulate their protein expression. These authors implicate miR-378b upregulation in the pathogenesis of ALD-associated hepatic insulin resistance.

There were several articles on the dysregulation of the gut-liver axis in ALD. Using both animal and clinical studies, the authors offered interventions to prevent the development of ALD. Zheng et al. demonstrated that treatment with *Lactobacillus reuteri* (*L. reuteri*) reversed the phenotype of ethanol-induced hepatitis and metabolic disorders in a mouse model of ALD. Their findings provided evidence that *L. reuteri* might serve as a new therapeutic strategy for ALD. Gao et al. presented a brief research report on the effect of 2-week of abstinence on the functional capacity of gut microbiota in patients with alcohol use disorder. Using a shotgun metagenomic sequencing, they identified different microbial functional responses to alcohol abstinence indicating a link between functional alterations of the gut microbiota and steatosis induced by alcohol consumption. These findings clearly lay foundation for a potential role of specific probiotic intake or fecal microbiota transplantation as an approach to treat alcohol use disorder. The next two articles demonstrated the importance of maintaining copper homeostasis and IFN-γ signaling to combat alcohol toxicity at the gut-liver axis, leading to a better control of the pathogenesis upon alcohol intoxication. While Lin et al. documented that copper maintains gut barrier integrity, possibly by regulating intestinal HIF-1α gene expression and oxidative stress, Yue et al. demonstrated that constitutive expression of IFN-γ is instrumental in maintaining intestinal STAT signaling, innate immune responses and gut microbial symbiosis. Yue et al. further suggested that IFN-γ-based interventions, such as IL-18 treatment, could be considered as a way of boosting intestinal epithelial innate immunity to halt systemic pathogen-associated molecular patterns translocation and may also be one approach for preventing the development of ALD. Tang et al. utilized a synthetic retinoid, fenretinide, and showed that it can be beneficial in preventing ALD, potentially through modulation of the intestinal barrier function, endotoxemia, and toll-like receptor 4-mediated inflammatory signaling. These authors in their brief research report suggested that pre-clinical investigations of this synthetic retinoid should be conducted as a potential pharmacological treatment for ALD.

A set of articles presented clinical findings and addressed ways to improve patient outcomes. A retrospective study presented by Waleed et al. on alcoholic hepatitis patients revealed a higher frequency of liver disease complications, hospital-acquired infections and longer duration of hospitalization in non-academic as compared to academic centers, despite similar hospitalization-related costs. The authors recommended conducting large prospective studies to validate these findings, examine mechanisms of infections in ALD patients and develop strategies to reduce hospital-acquired infections. A systematic review and meta-analysis by Hegyi et al. in patients with cirrhosis due to NASH showed that sarcopenic obesity is associated with two folds higher mortality at short and long-term follow up after receiving liver transplantation. This study suggested that the inclusion of body composition assessment before liver transplant may help to plan a more individualized nutritional treatment, physiotherapy, and postoperative care as a basis for improving the post-transplant outcomes of high risk obese patients with NASH who also have sarcopenia. Another clinical study by Riva et al. examined immunoregulatory checkpoint receptors in ALD patients vs. healthy controls. The authors reported that soluble T cell immunoglobulin and mucin domain-containing protein 3 (TIM3) was a dominant plasma checkpoint receptor in ALD patients, and this correlated with the disease severity. Increased TIM3 was also associated with elevated levels of soluble TIM3 ligands and membrane-TIM3 expression on immune cells. Importantly soluble TIM3 blocked the TIM3-ligand synapse to improve antibacterial immunity representing an innovative immune-based supportive treatment to rescue anti-bacterial defences in ALD patients. In the following clinical article Cheng et al. sought to characterize the sera metabolite fingerprints in ALD patients with and without ascites and found that ascites in ALD patients is closely associated with abnormalities in amino acid and lipid metabolism. The authors concluded that exploration of these metabolites be a novel biomarker or potential therapeutic targets in ALD patients. Neuman et al. in their article documented the persistence of characteristic features of ALD, namely centrilobular inflammation and programmed cell death, even after 1 week of alcohol detoxification despite improving various parameters such as liver stiffness and transaminase levels. They further reported that while apoptosis marker is generally higher in cirrhotic patients, its levels increased in response to withdrawal only in non-cirrhotic patients. These results suggested a role of liver micro-environment that, independent of amount of alcohol consumption, causes continued cell death by apoptosis. The authors concluded that further studies on heavy drinkers undergoing alcohol withdrawal may help to better understand molecular mechanism of liver damage and resolution in patients with ALD.

There were two manuscripts that compared alcohol-associated and non-alcohol-associated fatty liver diseases. A review paper by Zhang et al. summarized specific molecular mechanisms and intervention effectors for both these common liver diseases. The authors acknowledged that despite multiple common features such as lipid accumulation, oxidative stress, inflammation, insulin resistance, dietary habits, the mechanisms promoting disease progression is different which requires diversified treatment approaches. Along the same theme, Rasineni et al. using a rat model, showed that even though both alcohol-associated and non-alcohol-associated fatty liver diseases are nearly histologically indistinguishable, the physiological mechanisms that cause hepatic fat accumulation are different, as are their responses to starvation.

Several other articles in this Topic were solely devoted to the treatment/prevention of ALD. Lu et al. used both *in vitro* and *in vivo* models to illustrate that the protective effect of Qinggan Huoxue Recipe against alcohol-induced liver injury was by affecting lysophosphatidylcholine acyltransferase 3 and preventing endoplasmic reticulum stress. In their study, Chu et al. demonstrated that the leading mechanisms in liver injury development such as oxidative stress and pyroptosis were reversed by a phenolic acid compound, sinapic acid. The authors further showed that this compound is protective by potentially binding to bromodomain-containing protein 4, which functions as a transcriptional coactivator to carry out various pathophysiological activities. Another study by Arumugam et al. claimed that the therapeutic role of betaine in preventing liver injury in an animal model of binge drinking is *via* maintaining methylation potential. In addition to these preclinical studies, Yen et al. explored the clinical benefits of 12 weeks of treatment with antroquinonol extracted from a medicinal fungus, Golden-Antrodia camphorata, on hepatic function after alcohol consumption. As revealed, this agent suppressed ALT, AST, and triglyceride levels in patients with liver disease compared with the placebo group and was found to be safe for healthy subjects and patients with ALD.

Overall, this Research Topic covers important aspects on ALD and provides comprehensive updated information on mechanisms to better understand the disease pathogenesis and potential new biomarkers/therapeutic targets. The published articles are recommended for scientists and physicians involved in basic, translational and/or clinical studies on ALD. We strongly feel that the efforts of the guest editors and the journal in developing this Research Topic will be a small but significant step towards understanding, awareness, and hopefully reducing the disease burden of ALD, which is the most common liver disease worldwide and currently contributing to over 25% of cirrhosis related deaths.

